# Assessment of alcohol use disorder and its associated factors among alcohol users of medical and surgical outpatients attending a specialized hospital in Gondar, Ethiopia: a cross-sectional study

**DOI:** 10.1186/s13033-021-00454-2

**Published:** 2021-03-26

**Authors:** Demeke Demilew, Berhanu Boru, Getachew Tesfaw, Habtamu Kerebih, Endalamaw Salelew

**Affiliations:** 1 Department of Psychiatry, School of Medicine, College of Medicine and Health Sciences, University of Gondar, P.O.BOX: 196, Gondar, Ethiopia; 2 School of Nursing, College of Medicine and Health Sciences, University of Gondar, Gondar, Ethiopia

**Keywords:** Alcohol use disorder, Factors, Medical and surgical outpatients, Ethiopia

## Abstract

**Background:**

Alcohol use disorder increase the risk of physical harm, mental or social consequences for patients and others in the community. Studies on alcohol use disorder and associated factors among medical and surgical outpatients in Ethiopia are limited. Therefore, this study is meant to provide essential data on alcohol use disorder and associated factors among alcohol user medical and surgical outpatients to intervene in the future.

**Methods:**

An institution-based cross-sectional study was conducted by using the systematic random sampling technique. Alcohol use disorders were assessed using the World Health Organization’s 10-item Alcohol Use Disorder Identification Test (AUDIT) questionnaire. Bivariate and multivariate logistic regression analyses were performed, a P-value less than 0.05 were considered statistically significant in the multivariate analysis and the strength of association was measured at a 95% confidence interval.

**Results:**

The prevalence of alcohol use disorder was 34.5% with a 95% CI (29.20, 39.80) among study participants. In the multivariate logistic regression analysis, male sex (AOR = 3.33, 95%CI: 1.40, 7.93), history of mental illness (AOR = 2.68, 95%CI: 1.12, 6.38), drinking for relaxation (AOR = 1.88, 95%CI: 1.02, 3.48) and history of lifetime tobacco use (AOR = 5.64, 95%CI: 1.95, 16.29) were factors significantly associated with alcohol use disorder.

**Conclusion:**

The prevalence of alcohol use disorders among medical and surgical outpatients was found to be high. Male sex, history of mental illness, alcohol use for relaxation and lifetime cigarette smoking need more attention during the assessment of patients in the medical and surgical outpatient departments.

## Background

Alcohol use disorders (AUDs) can be classified as hazardous, harmful and/ or dependency[1]. The disorders affect the whole body system and has been implicated on liver disease, hypertension, myocardial disorders, immune dysfunction, neurological and psychiatric disorders, and other conditions [2]. Patients with AUDs are at increased risk for infections, wound, pulmonary complications, prolonged hospital stay and admissions into the intensive care unit after surgery. The problems lead many patients to poor surgical outcomes [3]. Alcohol causes cognitive impairment to the central nervous system with permanent structural damage to the brain, making people vulnerable to different psychiatric disorders and medical conditions [4]. The AUDs are potentially fatal that mimic and make worse a wide range of medical and psychiatric conditions, thereby shortening the life spans of affected people by more than a decade [5].

Despite its harsh and multifaceted effects, alcohol is the most widely consumed substance in the world. In 2004 World Health Organization (WHO) reports estimated that about two billion people worldwide were drinking alcoholic beverages, 76.3 million of whom had AUDs [6]. Globally, AUDs cause 2.5 million deaths each year, 80% of which occurred in a low income countries. The effects of the disorders accounted only for many of the deaths although it posed complications in various medical and surgical conditions [6, 7]. The prevalence of patients with AUDs identified in general hospitals was higher than the general population [8, 9]. The reason is that people with AUDs often seek help only when they become medically ill. However, in overcrowded clinical settings, medical staff often fail to recognize AUDs unless there are obvious physical or psychosocial effects relating to alcohol abuse [4].

Studies carried out in general hospital settings and in the community reported that different results in different countries. For instance, in the United States of America (USA) 20–53.5% [10–12], Nepal 7.5–40.5% [13, 14], Brazil 32.9–40% [2, 15], Taiwan Nigeria 9.7–41.4% [16, 17], South Africa 18.9% [18], and 34.8% [19], Uganda 4.1–17.4% [20, 21], and Ethiopia 3.0–32.6% [8, 9, 22, 23] had alcohol use disorders. Despite this high prevalence of AUDs in clinical settings, studies in Minnesota, USA and Twiwan revealed that less than a third of such individuals were identified as having alcohol use disorder among alcohol use disorders; (23.5%) by internists[24] and (6.5%) [24] by surgins and that only 10% and 5% of the patients were referred to psychiatric services for further evaluation and management respectively [24, 25]. The undetected alcohol use problems led to unpleasant consequences, like AUDs and which led to increase the morbidity and mortality of medical and surgical patients.

Many studies have revealed that younger age groups, the less-educated, a history of smoking, drinking fathers, male sex and getting treatment in internal medicine were factors significantly affecting AUDs [4, 26–28].

Evidences from previous studies showed that alcohol related problems among patient with general medical conditions posed greater consequences, including the worsening of the prognosis of the illness. The investigators, through their liaison services, observed a high number of patients with medical and surgical illnesses were consulted for psychiatric intervention due to alcohol withdrawal related problems. But, the magnitudes of alcohol use disorders among these groups of people have not been well explored in Ethiopia, particularly in the study area. These conditions led the investigators to assess alcohol use disorders and associated factors among patients treated for medical and surgical health problems. Therefore, the results of this study could be a vital input from health care providers to be vigilant of alcohol related problem early interventions and preventions of further complications.

## Methods and materials

### Study design, period and setting

An institution-based cross-sectional study was carried out from May 01 to 30, 2016. The study was conducted at the University of Gondar specialized hospital northwest Ethiopia. It is found in Gondar town, 727 km from Addis Ababa, the capital of Ethiopia. The hospital had 550 beds with around six medical and two surgical outpatient departments (OPDs), and gives a service for about five million people in the catchment area. In the last twelve months, the average adult outpatient flow to the medical and surgical outpatients was 2034 and 718 respectively.

### Population

The study samples were recruited among aged 18 years and above attendees to the medical and surgical outpatient departments. Severely ill, unable to hear, and having cognitive impairment were excluded**.**

### Sample size determination and technique

The sample size was determined using the single population proportion formula. So far, there has been no study on AUDs among surgical and medical outpatients in Ethiopia. Therefore, the initial the sample size was calculated by taking a 50% proportion at 95% level of confidence and a 5% margin of error. Assuming a 10% non-response rate, a total sample of 423 was obtained. To determine the maximum sample size, alternative sample size determination was done using the risk factors of alcohol use disorder. Previous studies indicate that male sex, use of cigarettes and khat are significantly associated with alcohol use disorder [8]. Based on these factors, sample size was determined with the following assumptions (Table 1).

**Table 1:** Sample size determination using predictors of alcohol use disorder

Factors associated with AUDs	Assumption used	Sample size with 10% non-respondent rate
Male sex	Odd Ratio (OR) = 2, 95%CI, Power = 80, P = 16	475
Cigarette smoking	OR = 2, 95%CI, Power = 80, P = 29.5	339
khat chewing	OR = 2, 95%CI, Power = 80, P = 28.72	343

Thus, a total sample of 475 was included in the study. Based on the twelve months average outpatient flow in each department, a proportional allocation was performed. Therefore, 351 and 124 patients were taken from the medical and surgical OPDs, respectively. The systematic random sampling technique was used to recruit each participant. Sampling interval was determined by dividing the number of the average monthly outpatients by the proportionally allocated outpatients to each department (k = 2034/351 = 6 to medical and k = 718/124 = 6 to surgical patients). Therefore, the participants were interviewed at every sixth regular interval, and the first participant was selected by the lottery method.

### Measurements

Data were collected by a face to face interviewers administered questionnaire and chart reviews conducted to know the types of diagnoses. The socio-demographic and related parts of the questionnaire were developed by reviews of literature. Alcohol use disorder was assessed by the WHO Alcohol Use Disorders Identification Test (AUDIT) screening tool. It is a primary and most effective tool to identify problematic alcohol use at an early stage with sensitivity 94.1% and specificity 91.7%[1]. The AUDIT comprises to address four areas; the first three questions (1–3) explore the quantity and frequency of alcohol consumption (Hazardous Alcohol Use); the second three questions (4–6) assessed signs of alcohol dependency while the last four (7–10) investigated alcohol-related problems (harmful alcohol use). Each question a response category that ranges from 0 to 4, with the first response for each question scoring “0” never, “1” less than monthly, “2” monthly’ “3” weekly, and “4” daily or almost daily. Question 9 and 10, have only three responses, and scored as 0, 2 and 4. The AUDIT are summed up to give an overall score ranging from 0 to 40. A total score of 0–7 indicated social drinking, a score of 8–15 indicated “hazardous drinking”, a score of 16–19 indicated “harmful drinking” and a score of 20 or above indicated probable alcohol dependence. The AUDIT score of eight or more was used to define probable AUDs. This alcohol screening tool has been validated across different African countries [1, 6, 29]. In previous works carried out in Ethiopia, the internal consistency of AUDIT was within the acceptable range (Cronbach’s α = 0.79) [9, 30]. The local made ethanol beverages, for example ‘Araki’ (distilled from fermented barley or maize combined with ‘gesho’ [Rhamnusprinioides]), ‘Tella’ (a beer like drink fermented from barley and Rhamnusprioides) and ‘Tej’ (honey-wine) were converted into standard drinking units based on their local measurement with an equivalent standard drinking measurement [8]. The data were collected by four trained bachelors of nursing graduates, supervised with one master’s in psychiatry. The total AUDIT score, alcohol consumption level, signs of dependence, and markers of present harm should play a role in determining how to manage a patient with AUDs [31]. In this study, the internal consistency of AUDIT was in the best range (Cronbach’s α = 0.94).

### Data quality control

The English version questionnaire was translated to Amharic and back to English by two different native language experts. A pre-test was conducted on 5% of the sample one week before the data collection at the Felegehiwot referral hospital, Bahir Dar, Ethiopia, to check understandability and reliability of the questionnaire. The supervisor and data collectors were trained in one day before the data collection on the objective of the study, and on how to handle ethical issues and the confidentiality of information.

### Statistical analysis

Data were checked for completeness, cleaned manually, pre-coded, entered into EPI info version 7 and exported to SPSS version 20 for further analysis. For descriptive variables, frequencies, percentages, graphs, and tables were used. In the binary logistic regression analysis, variables found to have a p-value of less than 0.2 were candidates for the multivariable logistic regression analysis. In the multivariable logistic regression analysis, variables with less than 0.05 p-values were considered as significantly associated with the outcome variable. The strength of associations was explained with an adjusted odds ratio (AOR) at 95% confidence interval (CI).

## Results

### Socio-demographic characteristics

A total of 470 patients participated in the study with a response rate of 98.95%. More than half of the participants, 255 (54.3%) were male, 256 (54.5%) urban residents, and the mean age of the respondents was 36.64 (SD $\pm$ 13.24) years. Out of the participants, 260 (55.3%), were married; 402 (85.5%) were Amhara, 436 (92.8%) Orthodox Christian followers 387 (82.3%) were living with their families and 147 (31.3%) were unable to read and write (Table 2).

**Table 2:** Distribution of sociodemographic characteristics of medical and surgical outpatients of University of Gondar specialized hospital, 2016 (n = 470)

Variables	Categories	Frequency	Percentage
Sex	Male	255	54.3
	Female	215	45.7
Age	18–24	106	22.6
	25–34	128	27.2
	35–44	90	19.1
	45–54	75	16.0
	55 +	71	15.1
Religion	Orthodox	436	92.8
	Muslim	27	5.7
	Protestant	4	0.9
	Others^R^	3	0.6
Ethnicity	Amhara	402	85.5
	Kimant	44	9.4
	Tigre	17	3.6
	Others^E^	7	1.5
Educational status	Unable to read and write	147	31.3
	Read and write only^a^	60	12.8
	Primary school (grade 1–8)	110	23.4
	Secondary school (grade 9–12)	76	16.2
	Tertiary(TVET/college/university)	77	16.4
Marital status	Single	135	28.7
	Married/in union	260	55.3
	Divorced	34	7.2
	Separated	11	2.3
	Widowed/ widower	30	6.4
Occupation	Government employee	59	12.6
	Housewife	102	21.7
	Merchant	57	12.1
	Daily laborer	20	4.3
	Student	57	12.1
	Farmer	122	26.0
	Others^O^	53	11.28
Residency	Urban	256	54.5
	Rural	214	45.5
Living conditions	With family	387	82.3
	Alone	83	17.7
Monthly income	< 735	229	48.7
	735–1176	95	20.2
	> 1176	146	31.1

^a^Indicate informally educated people

Others^R^ includes (Catholic, Johba witness, and Adventist)

Others^E^ includes (Agew, Benshangul, and Oromo)

Others^O^ includes (jobless, retired and carpenter)

### Clinical and substance use characteristics

Of the participants, 156 (33.2%) had a history of hospital admission; 210 (44.7%) had more than two years of illness; some 54 (11.5%), were diagnosed with extra pulmonary tuberculosis; 55 (11.7%) were diagnosed with diabetes mellitus and 73 (15.5%) with cardiovascular diseases. More than half of the participants, 244 (51.9%), had a family history of alcohol use; 29 (6.2%) were lifetime cigarette smokers and 11 (2.3%) current smokers. The most preferable types of alcohol used by the participants, 128 (39.8%) and 125 (38.8%) were Tella and beer/draft respectively (Table 3).

**Table 3:** Distribution of clinical, and substance use characteristics of medical and surgical outpatients at University of Gondar specialized hospital, 2016 (470)

Variables	Categories	Frequency (n = 470)	Percentage
Duration of illness	< 6 months	123	26.2
	6–24 months	137	29.1
	> 24 months	210	44.7
Previous hospital admission	Yes	156	33.2
	No	314	66.8
Type of OPD	Medical	350	74.5
	Surgical	120	25.5
History of mental illness	Yes	48	10.2
	No	422	89.8
Diagnosis	Tuberculosis	54	11.5
	Diabetes mellitus	56	11.9
	Cardiovascular	73	15.5
	Injury	29	6.2
	Tumor	38	8.1
	Gastritis	25	5.3
	Urinary tract infection	35	7.4
	Goiter	20	4.3
	Breast tumor	13	2.8
	Others^D^	127	27.0
Family history of alcohol use	Yes	244	51.9
	No	226	48.1
Lifetime use of cigarette smoking	Yes	29	6.2
	No	441	93.8
Lifetime use of khat	Yes	23	4.9
	No	447	95.1
Current use of cigarette smoking	Yes	11	2.3
	No	459	97.7
Current use of khat	Yes	15	3.2
	No	455	96.8
Current use of other substances	Yes	1	0.2
	No	469	99.8
Most preferable types of alcohol she/he drunk^a^	Tella	128	39.8
	Tej	4	1.2
	Araki	28	8.7
	Beer/draft	125	38.8
	Wine	12	3.7
	Whisky/Gin	25	7.8
Reason given for alcohol use	Socialization	244	75.8
	Peer pressure	56	17.4
	To relax	85	26.4
	To get relief from stress	13	4.0
	No specific reason	13	4.0

Others^D^ includes diagnosis (hypertension, leishmaniasis, dermatological cases, diarrhea cases, Pylonephritis, urolithiasis, typhus and typhoid, disc prolapsed, acute upper respiratory infection, parasitic infection, and other unspecified

^a^Local made ethanol beverages

### Magnitude of alcohol use and use disorders

The majority of the study participants, 322 (68.50%) had a history of alcohol use, of whom, 111 (34.5%) had alcohol use disorders as defined by AUDIT with a total cutoff point of eight and above with a 95% CI (29.20, 39.80); with 86 (26.7%), 16 (5%) and 9 (2.8%), hazardous drinking, harmful use, and dependent respectively. In this study, the prevalence of AUDs among men and women was 95 (85.6%) and 16 (14.4%), respectively. Of those, 75 (34.4%) medical outpatients and 36(34.6%) surgical outpatients had alcohol use disorders.

### Factors associated with alcohol use disorders

In the bivariate analysis, sex, educational status, diagnosis of injury, history of mental illness, occupation, lifetime tobacco use, peer pressure and drinking for relaxation were candidates for the multivariable logistic regression analysis at p-value < 0.2. In the multivariable logistic regression analysis, male sex, history of mental illness, drinking for relaxation and lifetime tobacco use were significantly associated with AUDs at p-value < 0.05.

The multivariate analysis suggested that male sex was 3.33 times more likely to develop AUDs compared to female sex (adjust odd ratio (AOR) = 3.33, 95%CI = 1.40, 7.93). Participants who had a history of mental illness were 2.68 times more likely risky to develop AUDs than those who had no such history (AOR = 2.68, 95%CI = 1.12, 6.38). Participants who drank for relaxation were 1.88 times more likely to have AUDs compared to participants who didn’t (AOR = 1.88, 95%CI = 1.02, 3.48) drink, and lifetime tobacco smokers were 5.64 times more likely to develop the problem than nonsmokers (AOR = 5.64, 95%CI = 1.95, 16.29) (Table 4).

**Table 4:** Bivariate and multivariate analysis of alcohol use disorders and associated factors among alcohol user medical and surgical outpatients at Gondar university hospital (n = 322), 2016

Variables	Categories	AUDs		OR (95% CI)	AOR (95% CI)
		Yes	No		
Sex	Female	16	101	1.00	1.00
	Male	95	110	5.45 (3.01,9.88)	3.33 (1.40,7.93)**
Educational status	Can’t read and write	31	75	1.18 (0.55, 2.51)	1.61 (0.46, 5.68)
	Read and write only	25	21	3.39 (1.44, 7.99)	2.94 (0.79, 10.94)
	Primary school(1–8)	31	43	2.05 (0.94, 4.49)	1.62 (0.52, 5.00)
	Secondary (9–12)	11	35	0.9 (0.35, 2.26)	0.93 (0.28, 3.03)
	Above grade, 12th	13	37	1.00	1.00
Mental illness history	No	94	198	1.00	1.00
	Yes	17	13	2.76 (1.29, 5.91)	2.68 (1.12, 6.38)*
Diagnosis of injury	No	97	199	1.00	1.00
	Yes	14	12	2.39 (1.07, 5.37)	2.06 (0.82, 5.15)
Drinking for relaxation	No	70	167	1.00	1.00
	Yes	41	44	2.22 (1.34, 3.70)	1.88 (1.02, 3.48)*
Lifetime use of tobacco	No	96	204	1.00	1.00
	Yes	15	7	4.56 (1.80,11.53)	5.64 (1.95, 16.3)**
Drinking due to peer pressure	No	86	180	1.00	1.00
	Yes	25	31	1.69 (0.94, 3.03)	1.26 (0.64, 2.50)
Occupation	Employed	11	28	1.00	1.00
	Housewife	8	48	0.42 (0.15, 1.18)	1.59 (0.33, 7.56)
	Merchant	13	20	1.66 (0.62, 4.44)	1.60 (0.47, 5.54)
	Daily laborer	6	7	2.18 (0.6, 7.96)	2.04 (0.42, 10.01)
	Student	9	32	0.72 (0.26, 1.98)	1.10 (0.34, 3.52)
	Farmer	50	58	2.19 (1.00, 4.85)	1.96 (0.63, 6.07)
	Others^O^	14	18	1.98 (0.74, 5.31)	2.37 (0.71, 7.88)

Significance * (p-value < 0.05), and ** (p-value < 0.01)

## Discussion

In this study, alcohol use disorders and associated factors among alcohol user medical and surgical outpatients were assessed. The result revealed that a remarkable proportion 34.5% of the participants had alcohol use disorders, at 95% CI (29.20, 39.80). Our finding was consistent with those of other studies for example, 32.6% in Ethiopia among HIV/AIDS outpatients [9], 34.8% in South Africa in urban primary hospital outpatients [19], and 32.9% in Brazil among hospitalized patients [2].

On the other hand, the findings of this study showed that much higher prevalence compared to many of the previous works carried out in different parts of the world which reported prevalence rates ranging from 4.1 to 27.6% [14, 17, 18, 20, 21, 32–37]. The possible reasons for the discrepancy might be variations in study populations, different measurement tools, study designs and the sociocultural practices of participants.

The result of the current study is higher compared to the previous studies conducted in the rural community of Ethiopia that reported a prevalence of ranging from 3 to 21% [8, 22, 23]. The possible explanations for the differences might be the production and usages of both locally made and industrially manufactured alcoholic drinks are higher in Gondar compared to those places where other researches were conducted. Moreover, the majority of the participants in our study were Orthodox-Christian followers in which alcohol consumption behavior has a more tolerant view. The other possible reason for variations might be the study populations. Because, the previous studies were a community survey conducted in the rural Ethiopia. The major causes of premature death among people with alcohol use disorders are injury, alcohol liver disease, heart disease, stroke, cancers, and gastrointestinal diseases and have greater use of health care resources [38]. Hence, that might be the possible reason for high prevalence among attendees at medical and surgical outpatient departments. Besides, the differences in findings also relate to the tools used. For instance, the Fast Alcohol Screening Test (FAST) was put to use in a study carried out in the rural Ethiopia [22].

On the other hand, the prevalence of alcohol use disorder in this work is lower than that of a study conducted on medical and surgical outpatients aged 45–64 years in Nigeria (41.4%) [16], 53.5% on America Veteran Affairs outpatients [12] and 40.5% in Nepal [13]. The possible reasons for the difference might be the tools. In Nigeria, for instance, a structured clinical interview Diagnostic Statistical Manual-IV was employed, while the International Classification of Disease-9 code for alcohol use disorder which has a high sensitivity for assessing such problems was used in America. When it comes to study populations; Veteran Affairs outpatients in America, while men in the 45–64 age group most prone to consume alcohol which is likely to increase prevalence were interviewed in Nigeria. In Nepal, only medical outpatients were considered. As a matter of fact, sociocultural variations are also responsible for differences in study results.

In our study, the prevalence of AUDs was 85.6% among men and 14.4% for women (male: female ratio 5.9:1), and slightly higher than those same epidemiological surveys in the United states 5:1 [39]. This showed that the prevalence rates were much narrower than was reported in China 66:1 [40]. This is as a result of that males are known to engage in risky behaviors, including heavy alcohol consumption, and this is consistent with several studies were done in Ethiopia and elsewhere, heavy drinking was associated with masculinity and is considered a male norm [9, 41, 42]. In this study, male sex was significantly associated with alcohol use disorders. The finding is supported by studies in Taiwan [27], the Republic of Ireland [3], Brazil [2], India [43], Tanzania [44], and Kenya [32]. The possible reason might be due to alcohol drinking behavior is more socially acceptable among males than females, and predisposing men to alcohol use disorders.

In the current study, history of smoking cigarette use was significantly associated with AUDs. The result is similar to findings in South Africa [18], Sri Lanka [45], and India [43]. The possible explanation might be that smokers used alcohol to stop the stimulation of the nicotine after they smoked.

History of mental illness was significantly associated with AUDs. The finding of our study is consistent with other studies reported in Ethiopia [22], Nigeria [17], and in South Africa [18]. Additionally, multiple population surveys have found that about many individuals who develop substance use disorders are diagnosed with mental disorders and vice versa [46, 47]. The possible explanation could be that, it is common to find people with mental disorder to be more prone to drink large amounts of alcohol possibly as self-treatment, but alcohol use disorders are associated with increased risk of developing mental illness. It’s difficult to determine causality, since this is a cross-sectional study. As a result, further study of a cause–effect relationship is recommended.

Participants who were using alcohol for relaxation were statistically significant predictors of AUDs. Psychological distress and stressful life events were risk factors for the use of alcohol a study reported in Sri Lanka [45]. Users of alcohol for relaxation have chances to increase the dose of alcohol to get the desired effect. Therefore, they are prone to develop alcohol use disorders.

## Limitations

The study is, we hope, effective in that it has used a standard tool for assessing the disorders. Despite its capacity to provide valid evidence, however, it has some limitations in that it is subject to social desirability bias. Besides, since the data were gathered by an interviewer administered questionnaire, respondents might have tended to reply in ways favorable to others by either under or over reporting. Moreover, the cross-sectional design we used has prevented us from reporting casual effect relationships. The other limitation is, the association of sociodemographic factors and the types of illnesses. That is the majority of the patients sought help for physical illness relating to AUDs rather than for the latter alone. Thus, further studies that include in-depth variables and other designs are needed.

## Conclusion

The prevalence of alcohol use disorders among medical and surgical outpatients was found to be high. The habit of screening and managing the problems in medical and surgical departments is low. The comorbidity of alcohol use problems with physical illnesses may affect the prognosis by further complicating physical illnesses due to diminishing the immunity and withdrawal effects. Therefore, collaborative consultation-liaison works between psychiatrists and other health professionals need to be strengthened. The training of clinicians on how to detect, manage and refer patients with alcohol withdrawal and use disorders is required. Moreover, male sex, history of mental illness, use of alcohol use for relaxation and tobacco smokers need further attention for the assessment of alcohol use disorder.

## Data Availability

The data and materials used in this study are available from the corresponding author on a reasonable request.

## Abbreviations

AMSH: Amanuel Mental Specialized Hospital
ASSIST: Alcohol, smoking and substance involvement screening test
AUDs: Alcohol use disorders
AUDIT: Alcohol use disorders identification test
DSM-IV: Diagnostic and Statistical Manual of Mental Disorder 4 Edition
CAGE: Cut-annoyed-guilty- eye opener
OPD: Out Patient Department
SPSS: Statistical Package for the Social Sciences
WHO: World Health Organization
